# Oxidative Stress in the Muscles of the Fish Nile Tilapia Caused by Zinc Oxide Nanoparticles and Its Modulation by Vitamins C and E

**DOI:** 10.1155/2018/6926712

**Published:** 2018-04-05

**Authors:** Aaser M. Abdelazim, Islam M. Saadeldin, Ayman Abdel-Aziz Swelum, Mohamed M. Afifi, Ali Alkaladi

**Affiliations:** ^1^Department of Biochemistry, Faculty of Veterinary Medicine, Zagazig University, Zagazig 44519, Egypt; ^2^Department of Basic Medical Sciences, College of Applied Medical Sciences, University of Bisha, Bisha, Saudi Arabia; ^3^Department of Animal Production, College of Food and Agricultural Science, King Saud University, Riyadh 11451, Saudi Arabia; ^4^Department of Physiology, Faculty of Veterinary Medicine, Zagazig University, Zagazig 44519, Egypt; ^5^Department of Theriogenology, Faculty of Veterinary Medicine, Zagazig University, Zagazig 44519, Egypt; ^6^Department of Biological Science, Faculty of Science, University of Jeddah, Jeddah, Saudi Arabia; ^7^University of Jeddah Center for Scientific and Medical Research, University of Jeddah, Jeddah, Saudi Arabia

## Abstract

The effects of zinc oxide nanoparticles (ZnONPs) on antioxidants in Nile tilapia muscles and the protective role of vitamins C and E were examined. Two hundred males of Nile tilapia were held in aquaria (10 fishes/aquarium). Fishes were divided into 5 groups: 40 fishes in each group; the first group was the control; the 2nd and 3rd groups were exposed to 1 and 2 mg/L of ZnONPs, respectively; and the 4th and 5th group were exposed to 1 and 2 mg/L of ZnONPs and treated with a (500 mg/kg diet) mixture of vitamin C and E mixture (250 mg/kg diet of each). Muscles were collected on the 7th and 15th day of treatments. Muscle malondialdehyde, reduced glutathione levels, superoxide dismutase (SOD), catalase (CAT), reduced glutathione (GR), glutathione peroxidase (GPx), and glutathione-*S*-transferase (GST) activities were measured after treatments. Relative quantification of SOD, CAT, GR, GPx, and GST mRNA transcripts was detected in the muscles. Results showed that MDA and GSH concentration; SOD, CAT, GR, GPx, and GST activities; and mRNA expression were significantly decreased in groups exposed to ZnONPs. Vitamins C and E significantly ameliorated the toxic effects of ZnONPs. In conclusion, vitamins C and E have the ability to ameliorate ZnONP oxidative stress toxicity in Nile tilapia.

## 1. Introduction

Despite their useful properties, nanoparticle hazards on the biological system are poorly understood up till now [1–3]. The wide production and huge use of nanoparticles facilitate their possibility to induce hazards, for example, the use of nanoparticles in water waste treatment leads to their spread in the aquatic environment inducing a huge hazard for both human and aquatic beings [4]. The hazards of nanoparticles (NPs) generally and zinc oxide nanoparticles (ZnONPs) especially in aquatic environment may be related to their ability to induce an oxidative stress [5]. Furthermore, the generation of reactive oxygen species (ROS) could be influenced by the size and shape of NPs as well as the experimental conditions [6]. Several studies have examined the effect of ZnONPs on aquatic environment. Cytotoxicity and genotoxicity on the freshwater molluscan bivalve *Coelatura aegyptiaca* have been approved [7]. Moreover, the embryotoxicity of ZnONPs to marine medaka, *Oryzias melastigma*, was explored [8]. Additionally, changes in the transcriptional profile in larval zebrafish exposed to ZnONPs have been reported [9]. Now, there is a great concern that ZnONPs have a powerful effect on the aquatic environment and organisms. Antioxidant enzymes have been used as a biomarker for detection of contamination of Nile tilapia and its possibility to be potential candidates for tissue toxicity biomarkers of pollutants [10]; also, they have been used as biochemical markers for short-term exposure to diesel oil, pure biodiesel, and biodiesel blends in Nile tilapia [11]. This gave us the light to examine these antioxidant enzymes as targets for ZnONP exposure in Nile tilapia (*Oreochromis niloticus*). We especially chose Nile tilapia for our study due to its importance as an economical source of food and its varied systems of cultivation in developed countries [12]. The activity and expression profile of antioxidant enzymes under the effect of ZnONPs as well as the protective role of vitamins C and E were studied. We preferred to examine both vitamin C and E due to their important antioxidant capacity. The importance of vitamin C on fish health has been approved [13], while on the other hand, vitamin E has been used to control cyanotoxin [14], and both vitamin E and C have been used to control the hazards of metal toxicity in Nile tilapia [15]. At this moment, there is unclear role for both vitamin E and C to compete the toxicity originated form NP exposure. Only one recent study has showed the protective role of vitamin E alone against exposure to ZnONPs in Nile tilapia [16]. This gives us the impetus to validate their role to overcome the ZnONP toxicity on tilapia.

## 2. Material and Methods

### 2.1. Fish Preparation and Management

Two hundred males of *O*. *niloticus*, weight 90 ± 5 g, length 15 ± 3 cm, were obtained from Abrahem El-Solimani farms for fish, Kholes, KSA. The fishes were held in twenty glass boxes (*n* = 10 individuals/box), with 100 liters of water (pH 7.16 ± 0.3, 0.52 mM CaCl_2_, and 0.24 mM MgCl_2_) that was changed daily, a continuous system of water aeration (Eheim Liberty 150 Bio-Espumador cartridges). Temperature was maintained at 28 ± 2°C and O_2_ at 7.0 ± 0.5 mg/L. Fishes were fed on fish diet containing proteins (31%), carbohydrates (37%), lipids (6%), fibers (2.5%), total phosphorus (1.5%), ash (12%), *α*-tocopherol (200 mg/kg diet), vitamin D3 (1700 IU/kg diet), and vitamin A (10,000 IU/kg diet). Daily change of water was established to eliminate any residuals of food or NPs. Institutional and national guidelines for the care and use of fisheries were followed.

### 2.2. Fish Grouping and Induction of ZnONP Toxicity

The fishes were randomly divided into 5 groups, 40 fishes in each group triplicate; the first group served as control (C) and the 2nd and 3rd groups were exposed to ZnONPs of 1 and 2 mg/L (T1 and T2), respectively. This dosage was determined in review of the related literature [17, 18]. The 4th and 5th group were exposed to ZnONPs of 1 and 2 mg/L and treated with a mixture of vitamins C and E in a dose of 500 mg/kg diet (250 mg of each), T1 + V and T2 + V, respectively. After 7 and 15 days of exposure, there was no mortality, and twenty fishes of each group were randomly selected and were anesthetized on ice. The muscles were removed, frozen in liquid nitrogen, and stored at −80°C until experimental procedures.

### 2.3. Preparation and Characterization of ZnONP Particle Suspensions

**Figure 1:**
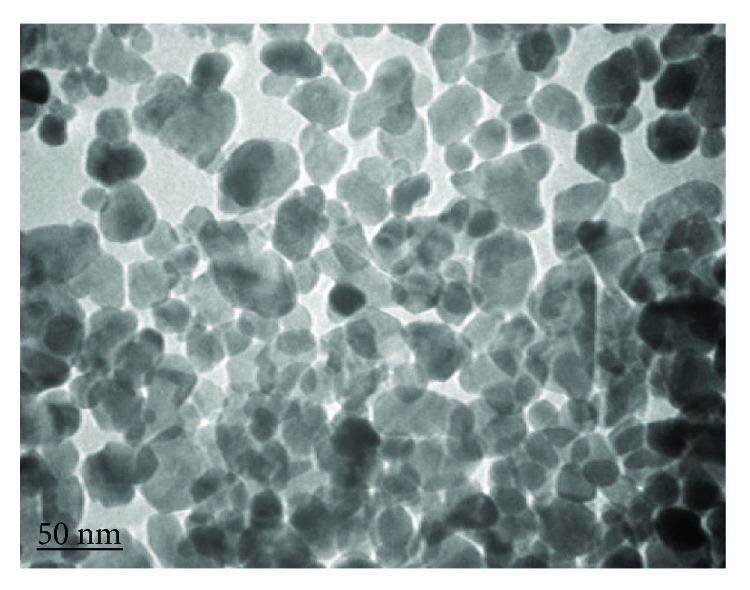
Transmission electron microscopic (TEM) photomicrograph of ZnONPs, which shows that the APS is 30 ± 5 nm.

ZnONPs were obtained in the form of dispersion from Sigma-Aldrich, Steinheim, Germany (CAS number 1314-13-2), of concentration 50 wt.% in H_2_O; average particle size (APS) was <35 nm Figure 1. The particle size distribution (hydrodynamic diameter) was <100 nm using dynamic light scattering (DLS) technique, pH 7 ± 0.1(for aqueous systems), and density 1.7 ± 0.1 g mL^−1^ at 25°C. Suspensions of ZnONPs in a concentration of 1 and 2 mg/L were daily prepared (JL-360, Shanghai, USA) for 20 min. To characterize the ZnONP shape and size, a small drop of aqueous ZnONP solution was air dried by directly placing onto a mesh of carbon-coated copper grid then examined under transmission electron microscope (TEM) (JEM-1011, JEOL, Japan). The concentration of ZnONPs in the exposure solution was quantified by inductively coupled plasma mass spectrometry (ICP-MS) at zero, 12, and 24 h of exposure to verify the exposure concentration is the same as the prepared concentrations (Supplemental Table 1).

### 2.4. Tissue Preparations

Trunk muscle homogenate was prepared from each sample without pooling according to [19], where 0.5 g of each muscle homogenization was performed using a solution formed from 5 mL of 0.1 M potassium phosphate buffer (pH 6.5) containing 20% (*v*/*v*) glycerol, 1 mM EDTA, and 1.4 mM dithioerythritol, centrifuged at 3000 rpm/5 m; then, the supernatant was used for biochemical assays.

### 2.5. Malondialdehyde (MDA), Reduced Glutathione (GSH), and Antioxidant Enzyme Activity Analysis

The biochemical levels of MDA [20] and GSH [21] and the activities of SOD [22], CAT [23], GR [24], GPx [25], and GST [26] were determined in the muscles of all experimental fishes. Details about the biochemical analysis were included in Supplemental Methods.

### 2.6. Antioxidant Enzyme mRNA Expression Levels by RT-PCR

Muscle SOD, CAT, GPx, GR, and GST gene expression was quantified using real-time PCR. RNA was isolated from the muscles using the RNeasy Mini Kit (Qiagen) (Cat. number 74104). 0.5 *μ*g of total RNA was used for production of cDNA using Qiagen Long Range 2 Step RT-PCR Kit (Cat. number 205920). Five *μ*L of total cDNA was mixed with 12.5 *μ*L of 2x SYBR® Green PCR mix with ROX from BioRad and 10 pmol/*μ*L of each forward and reverse primer for the measured genes. Primer3 software was used for primer design (The Whitehead Institute, http://bioinfo.ut.ee/primer3-0.4.0/) as per the published *O*. *niloticus* SOD, CAT, GPx, GR, GST, and *β*-actin gene sequences (JF801727.1, JF801726.1, FF280316.1, XM_003445184, EU234530, and EF206801), respectively, of NCBI database; all primers were provided by Sigma-Aldrich (Sigma-Aldrich Chemie GmbH, Steinheim, Germany) and are shown in Table 1. PCR reactions were carried out in a thermal cycler (AbiPrism 7300) (Applied Biosystems, USA). The quantitative fold increase in genes was determined in relation to *β*-actin mRNA gene and calculated by the 2^−DD CT^ method.

### 2.7. Statistical Analysis

Statistical Package for Social Science (SPSS Inc., Chicago, IL, version 20, USA) was used to analyze all data. The data were expressed as mean ± SD. One-way analysis of variance (ANOVA) was used to a comparison among groups. For intergrouping homogeneity, Duncan's test was used. Statistical significance was set at *P* ≤ 0.05.

## 3. Results

### 3.1. Effects of ZnONPs on Antioxidant Enzyme Activities and Gene Expression in Fish Muscles

**Figure 2:**
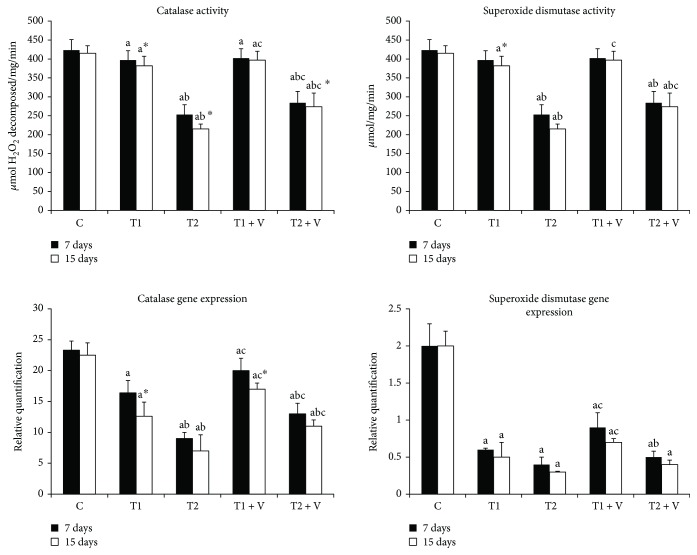
Activities and mRNA expression of muscle antioxidant enzymes, catalase, and superoxide dismutase in the (C) control group and in those (T1) exposed to 1 mg/L of zinc oxide nanoparticles, (T2) exposed to 1 mg/L of zinc oxide nanoparticles, (T1 + V) exposed to 1 mg/L of zinc oxide nanoparticles with a mixture of both vitamin C and E, and (T2 + V) exposed to 2 mg/L of zinc oxide nanoparticles with a mixture of both vitamin C and E. Values are expressed as mean ± SD (*n* = 20). Significant levels (*p* < 0.05) observed are as follows: a = in comparison to control group, b = when 2 mg ZnONP groups versus 1 mg ZnONP groups are compared, and c = when ZnONPs + vitamin groups versus their respective ZnONP groups are compared. ^∗^When 15-day-treated groups are compared with their respective 7-day-treated groups.

**Figure 3 fig3:**
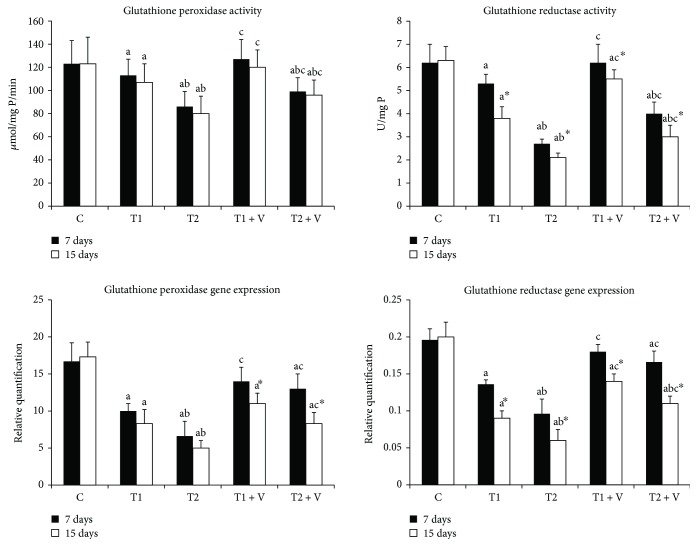
Activities and mRNA expression of muscle antioxidant enzymes glutathione peroxidase and glutathione reductase in the (C) control group and in those (T1) exposed to 1 mg/L of zinc oxide nanoparticles, (T2) exposed to 1 mg/L of zinc oxide nanoparticles, (T1 + V) exposed to 1 mg/L of zinc oxide nanoparticles with a mixture of both vitamin C and E, and (T2 + V) exposed to 2 mg/L of zinc oxide nanoparticles with a mixture of both vitamin C and E. Values are expressed as mean ± SD (*n* = 20). Significant levels (*p* < 0.05) observed are as follows: a = in comparison to control group, b = when 2 mg ZnONP groups versus 1 mg ZnONP groups are compared, and c = when ZnONPs + vitamin groups versus their respective ZnONP groups are compared. ^∗^When 15-day-treated groups are compared with their respective 7-day-treated groups.

**Figure 4:**
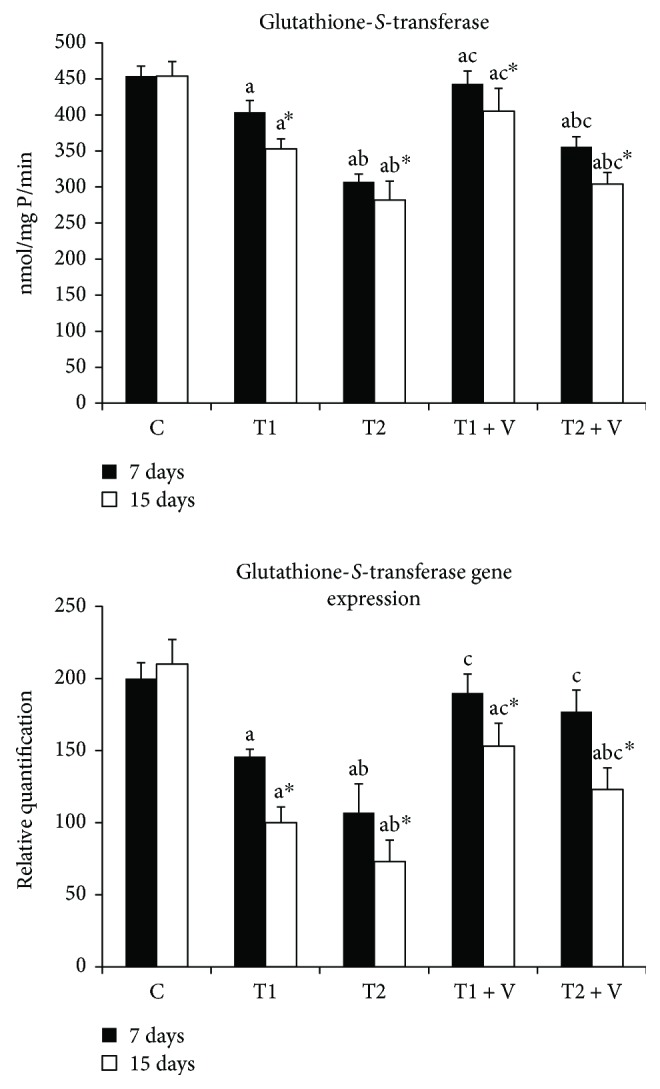
Activities and mRNA expression of muscle antioxidant enzymes glutathione-*S*-transferase in the (C) control group and those (T1) exposed to 1 mg/L of zinc oxide nanoparticles, (T2) exposed to 1 mg/L of zinc oxide nanoparticles, (T1+V) exposed to 1 mg/L of zinc oxide nanoparticles with a mixture of both vitamin C and E, and (T2+V) exposed to 2 mg/L of zinc oxide nanoparticles with a mixture of both vitamin C and E. Values are expressed as mean ± SD (*n*=20). Significant levels (*p* < 0.05) observed are as follows: a = in comparison to control group, b = when 2 mg ZnONP groups versus 1 mg ZnONPs groups are compared, and c = when ZnONPs + vitamin groups versus their respective ZnONPs groups are compared. ^∗^When 15-day-treated groups are compared with their respective 7-day-treated groups.

The activities of antioxidant enzymes in the muscle tissues are shown in Figures 2 [Fig fig3]–4. ZnONPs significantly inhibited CAT, SOD (Figure 2), GPx, GR (Figure 3), and GST (Figure 4) activities in muscles of ZnONP-exposed groups when compared to their control (*p* < 0.05). Supplementation of vitamin C and E mixture significantly ameliorated the enzyme activities to a similar level when compared with their control of nontreated fishes (*p* < 0.05). ZnONPs induced a significant repression of the relative mRNA expression of SOD and CAT (Figure 2), GPx and GR (Figure 3), and GST (Figure 4) in the muscles of exposed groups when compared with their control (*p* < 0.05). Supplementation of vitamin C and E mixture caused a significant induction in the antioxidant enzyme relative mRNA expression in muscles if compared with exposed nontreated fishes (*p* < 0.05).

### 3.2. Effects of ZnONPs on the Level of Reduced Glutathione (GSH) and MDA in Fish Muscles

**Figure 5:**
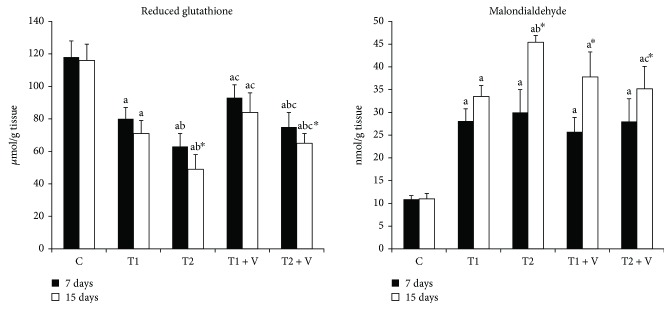
Level of malondialdehyde and reduced glutathione in the (C) control group and those (T1) exposed to 1 mg/L of zinc oxide nanoparticles, (T2) exposed to 1 mg/L of zinc oxide nanoparticles, (T1+V) exposed to 1 mg/L of zinc oxide nanoparticles with a mixture of both vitamin C and E, and (T2+V) exposed to 2 mg/L of zinc oxide nanoparticles with a mixture of both vitamin C and E. Values are expressed as mean ± SD (*n* = 5). Significant levels (*p* < 0.05) observed are as follows: a = in comparison to control group, b = when 2 mg ZnONP groups versus 1 mg ZnONP groups are compared, and c = when ZnONPs + vitamin groups versus their respective ZnONP groups are compared. ^∗^When 15-day-treated groups are compared with their respective 7-day-treated groups.

ZnONPs induced a significant decline in the concentration of GSH (Figure 5) in the muscles of exposed groups when compared with their control. Supplementation of vitamins C and E induced an increase in its level in exposed groups when compared with nontreated groups. ZnONPs also induce a significant increase in the concentration of MDA (Figure 5) in the muscles of exposed fishes when compared with their control. Supplementation of vitamins C and E did not induce significant changes of MDA in exposed groups when compared with the nontreated groups. In the same time, increase in the duration of exposure leads to increase of the generation of MDA in the muscles of exposed fishes.

## 4. Discussion

In the present study, we evaluated the possible effect of ZnONPs on the antioxidant system in *Oreochromis niloticus*. We also validated the use of antioxidants as bioindicators for NP exposure. The high exposure of humans and animals to NPs is the main subject that motivates us to do this work. There is great importance for such study. First, to the best of our knowledge, this is the first record about the sublethal effect of ZnONPs in Nile tilapia. Second, it is important to assess the oxidative stress risk resulted from ZnONP exposure, not only in aquatic organisms but also for human and all consummated beings. Third, we aimed to evaluate the possible protective role of vitamins C and E for of the ZnONP exposure drawbacks. The minimal effective dose (1 mg/L) of ZnONPs was used and it was duplicated (2 mg/L) for intensifying their effects. We selected muscles as a target for our study as it has been approved to be the main site for NPs deposition [5]. Moreover, muscles are the main consumed part of fish so their effect will directly reflected on human health.

### 4.1. Effects of ZnONPs on Antioxidant Enzyme Activities, Glutathione, and Lipid Peroxide Levels in Fish Muscles

The current study has showed a great effect of ZnONPs on the antioxidant enzyme activities and their mRNA expression in the muscles of *O*. *niloticus*. In general, NPs can induce their toxicity through many mechanisms; much of NPs have an oxidant power through production of reactive oxygen species (ROS) or through its power to inhibit cells antioxidant system [27, 28]. Our results showed a decline in superoxide dismutase (SOD), catalase (CAT), glutathione peroxidase (GPx), glutathione reductase (GR), and glutathione-*S*-transferase (GST) activities, respectively, in ZnONP-exposed groups. Therefore, our results could indicate the involvement of oxidative stress in response to ZnONPs. The concept of involvement of ZnONPs in the oxidative stress development has been recently approved in other organisms that have been exposed to NPs rather than *O*. *niloticus* [7, 29–36]. All these recent observations strengthen our concept of the involvement of ZnONPs in elevation of oxidative stress in fishes. Furthermore, our results showed that ZnONP effects were directly proportional to the period of exposure and in a dose-dependent manner.

#### 4.1.1. Effect of ZnONP Exposure on SOD and CAT Activities and mRNA Expression Levels

SOD and CAT are used as potent markers for early detection of environmental oxidative pollution; their activity was significantly reduced in ZnONP-exposed groups. The reduction of SOD and CAT activities and their mRNA expression has been used as an indicator for oxidant eradication [37]. In the same line of our study, the same observations were found in Chinook salmon cells exposed to 10–60 *μ*g/mL of titanium oxide NPs [38]; in the brain of *O*. *niloticus* and *Tilapia zilli* exposed to 2 and 4 mg/L of silver NPs [39]; in the liver of adult Japanese medaka exposed to nanoiron [40]; in Mozambique tilapia, *Oreochromis mossambicus*, exposed to nickel NPs [41]; in heart and gill cell lines of *Catla catla* and gill cell line of *Labeo rohita* exposed to silver NPs [42]; and in *Carassius auratus* exposed to 20, 40, 80, 160, and 230 mg/L of a mixture of copper NPs, zinc oxide NPs, and cerium oxide NPs and individual NP, respectively [43], while our results regarding SOD and CAT activities have come in contrary to previous studies conducted on a freshwater fish, *Carassius auratus*, livers and gills exposed to 10 *μ*g/mL of ZnONPs [44] and Chinook salmon cells exposed to 10–60 *μ*g/mL of copper oxide NPs [45].

#### 4.1.2. Effect of ZnONP Exposure on GPx, GR, and GST Activities and GSH Levels and mRNA Expression Levels

Our results showed a significant decline in GPx, GR, and GST activities and their mRNA expression levels in ZnONP-exposed groups. Previous studies have showed the same observations regarding these enzyme activities and/or mRNA expression levels. The effect of 2 and 4 mg/L of silver NPs on *O*. *niloticus and T*. *zillii* was studied; the GPx, GR, and GST activities and expression have been declined in the fish brain [39]. The same results have been obtained in the cells of Chinook salmon exposed to 10–60 *μ*g/mL of copper oxide NPs [45]. In the same line, GST was declined in the livers of *Carassius auratus and Danio rerio* following exposure to 0, 0.01, 0.1, 1, 10, and 100 mg/L of titanium oxide NPs [46]. From the previously mentioned, we can say that antioxidant enzyme activities have tended to be increased after a short time of NP exposure, while longer time of exposure can lead to inhibition of their activities and moreover their production. In our study, we have observed that the activities of enzymes at 7-day exposure period were higher than the activities after 14-day exposure. Our data come in the same line of the observations of [44]. The activities of GPx, GR, and GST were directly proportional to the GSH levels. This was also clear from our data, which has showed a low level of muscle GSH in the exposed groups. Usually, the level of GSH is directly proportional to GPx, GR, and GST activities, and this has been approved in the previous studies [5, 18, 38, 47–51].

#### 4.1.3. Effect of ZnONP Exposure on Malondialdehyde (MDA) Levels

Our results showed high levels of lipid MDA in the muscles of exposed fishes. The levels of malondialdehyde (MDA) as a potent marker for LPOs have been used in the present study. In the same line of our data, other authors also have proved high generation of MDA under ZnONP exposure [44, 52] and under other NP exposure [48, 50, 53]. In general, MDA has been considered a potent indicator for oxidative stress generation; the approved ability of ZnONPs to generate an oxidative stress has led directly to the increase of MDA levels in the tissues of the affected organism. The level of MDA was usually increased with the increase of exposure duration, and this was approved in our study. We can establish a concept here; the ability of NPs to induce toxicity to aquatic organisms depended on their doses, the duration of exposure, and their accessibility and distribution in organisms' tissues. Authors have approved that the gills, livers, brains, and muscles were the most affected fish parts when they are exposed to NPs. From our point of view, the muscles were considered the most important tissues, as they are the most consumed tissue by human beings. ZnONPs have induced an oxidative stress in the muscles of Nile tilapia, and alleviating this toxic effect is an important issue.

### 4.2. Effect of Vitamins C and E as Protective Agents for ZnONP Exposure

Vitamin C scavenged superoxide anion by forming semidehydroascorbate radical which is subsequently reduced by GSH [54], while vitamin E has the ability to stop lipid peroxidation in the cell membrane. It does this by two ways: first by unsaturated fatty acid interaction and second by preserving the protein peptide chains [55]. In addition, it scavenges O_2_, H_2_O_2_, (OH^−^) radicals, and (O^−^) radicals. The ability of both vitamin C and E to overcome the oxidative stress generated in the cells has now become a clear issue. Also, both vitamins have been known to have active pharmacological action, and they were therapeutically used in treatment of many oxidative stress-based diseases [56]. However, the challenge here is as follows: are they able to overcome the oxidative stress generated from exposure to sublethal doses of NPs? In the present study, we examined this. The results showed that there was a significant neutralization in the antioxidant system; the activities of SOD, CAT, GPx, GR, and GST started to return to normal as control with amelioration in the GSH levels and reduction in LPOs was observed in all fishes fed vitamin E and C mixture. This confirms the ability of both vitamin C and E to fight the oxidative damage results from ZnONP exposure. Our results were confirmed by previous observations; recently, vitamin C has been used to prepare a ligand for cerium oxide NPs as promising tool for facilitating NP detections in tissues [57]. This explained how much it has the power for binding of NPs; this illustrated the efficacy of vitamin C to tight bind to NPs and do its action. In the same line of our study, vitamin C and E mixture have the ability to correct some hematological and biochemical disorders in *O*. *niloticus* exposed to sublethal doses of ZnONPs [58]. In addition, vitamin E and vitamin C have induced protection against the cytotoxicity of silicon NPs [59]. This come in the same line of our finding. Moreover, the existence of NPs themselves could increase the activities of both vitamin C and E [60].

In conclusion, sublethal doses of ZnONPs were able to induce an oxidative stress in the muscles of *O*. *niloticus*, and a mixture of vitamins C and E was able to alleviate the oxidative stress generated due to exposure to ZnONPs.

## Figures and Tables

**Table 1:** Oligonucleotide sequences of primers for examined antioxidant enzymes.

Gene	Forward 5′->3′	Reverse 5′->3′	Amplicon size (pb)
CAT	tcctgaatgaggaggagcga	atcttagatgaggcggtgatg	**232**
SOD	ggtgccctggagcccta	atgcgaagtcttccactgtc	**377**
GPx	ccaagagaactgcaagaga	caggacacgtcattcctacac	**180**
GR	cattaccgagacgcggagtt	cagttggctcaggatcatttgt	**420**
GST	taatgggagagggaagatgg	ctctgcgatgtaattcagga	**640**
*β*-actin	caatgagaggttccgttgc	aggattccataccaaggaagg	**280**

## Supplementary Materials

Supplementary 1 Supplemental Table 1: the actual ZnONP concentrations (mg/L) in the exposure water.

Supplementary 2 Supplemental Methods (Biochemical assays).
