# Supplementary material for: Oxidative Stress in the Muscles of the Fish Nile Tilapia Caused by Zinc Oxide Nanoparticles and Its Modulation by Vitamins C and E
Source: Oxid Med Cell Longev. 2018 Apr 5;2018:6926712. doi: 10.1155/2018/6926712 (PMC5907420; doi:10.1155/2018/6926712)
Supplement: Supplementary 1 — Supplemental Table 1: the actual ZnONP concentrations (mg/L) in the exposure water. [file 6926712.f1.pdf]

**Supplemental Table (1):** The actual ZnONPs concentrations (mg/ L) in the exposure water.

| **Concentrations (mg/ L)** | **Time (hours)** | | |
|---|---|---|---|
| | Zero | 12 | 24 |
| **1$^{st}$ group** | Nd | Nd | Nd |
| **2$^{nd}$ group** | 1±0.003 | 0.96±0.003 | 0.92±0.001 |
| **3$^{rd}$ group** | 2±0.005 | 1.98±0.006 | 1.95±0.004 |
| **4$^{th}$ group** | 1±0.003 | 0.93±0.003 | 0.90±0.001 |
| **5$^{th}$ group** | 2±0.004 | 1.96±0.006 | 1.93±0.004 |

Nd= not detected
