# Supplementary material for: Oxidative Stress in the Muscles of the Fish Nile Tilapia Caused by Zinc Oxide Nanoparticles and Its Modulation by Vitamins C and E
Source: Oxid Med Cell Longev. 2018 Apr 5;2018:6926712. doi: 10.1155/2018/6926712 (PMC5907420; doi:10.1155/2018/6926712)
Supplement: Supplementary 2 — Supplemental Methods (Biochemical assays). [file 6926712.f2.docx]

**Supplemental Methods (Biochemical assays)**

***(1) MDA assay*** (Gasparovic et al. 2013)

Muscles were isolated washed by PBS before their homogenization, homogenized tissues were mixed with lysis buffer then centrifuged for collection of supernatant, then TBA was added to both supernatant samples and standard, boiled in water bath then cooled for 10 minutes, 200 µL of each sample and standard are picked and measured color metrically.

***(2) GSH assay*** (Esterbauer &Cheeseman 1990)

Reaction mix containing NADPH, GR and glutathione buffer was prepared. Samples and standard were added to the reaction mix, then incubation for 10 minutes. The substrate is then added and the OD is determined colormeterically at OD 405 nm.

***(3) CAT assay*** (Paoletti &Mocali 1990)

Muscles were homogenized in PBS and EDTA for removal of blood debris then centrifuged. Supernatant was obtained on ice and unused samples were stored at -70 °C. Standard curve was prepared. 200 µL of each sample and standard were added to 500 µL of H_2_O_2_ working reagent, mixed and incubated for one minute. The reaction was stopped by adding Catalase quencher. 500 µL of coloring working reagent were added for each sample and standard and incubated for 30 minutes. OD was measured at OD520nm.

***(4) SOD assay*** (Aebi, 1984)

Muscles were prepared, homogenized in lysis buffer containing Tris, NaCl and EDTA. Then a master mix containing chromgen, Xanthine solutions, 10X SOD buffer was mixed with samples and standard. Xanthine oxidase was added and incubated for 1 hour then OD measured at 490 nm.

***(5) GPx assay*** (Carlberg and Mannervik, 1985)

Muscles were collected washed with PBS, homogenized and resuspended in the assay buffer. Samples and standard were added to a reaction mix containing (assay buffer, NADPH, GR solution and GSH solution), mixed and then incubated at 25 °C for 15 minutes. Cumene hydroperoxide solution was added to start GPx reaction. OD1 was measured at 340 nm. The reaction was further incubated at 25 °C for 5 minutes then OD2 was measured. ΔOD was then measured.

***(6) GR assay*** (Beutler 1969)

Muscles were washed in PBS and homogenized in a buffer solution containing potassium phosphate and EDTA). Supernatant was obtained for biochemical assay. Samples and standard were mixed with assay buffer and GSSG for few seconds. NADPH was then added to start the reaction. OD is determined at 340 nm every minute. Then ΔOD is calculated and the activity was calculated according to the standard curve.

***(7) GST assay*** (Wilce and Parker, 1994)

Samples were prepared and homogenized in PBS. The master mix containing PBS, GSH and substrate was added to the sample. OD was measured every minute for 5 minutes then ΔOD is calculated at 340 nm. The activity is calculated according to the standard curve.
